# Plant rDNA database: update and new features

**DOI:** 10.1093/database/bau063

**Published:** 2014-06-28

**Authors:** Sònia Garcia, Francisco Gálvez, Airy Gras, Aleš Kovařík, Teresa Garnatje

**Affiliations:** ^1^Laboratori de Botànica-Unitat associada CSIC, Facultat de Farmàcia, Universitat de Barcelona, Barcelona, 08028 Catalonia, Spain, ^2^BioScripts – Centro de Investigación y Desarrollo de Recursos Científicos, Sevilla, 41012 Andalusia, Spain, ^3^Institute of Biophysics, Academy of Sciences of the Czech Republic. Brno, CZ-612 65, Czech Republic and ^4^Institut Botànic de Barcelona (IBB-CSIC-ICUB). Barcelona, 08038 Catalonia, Spain

## Abstract

The Plant rDNA database (www.plantrdnadatabase.com) is an open access online resource providing detailed information on numbers, structures and positions of 5S and 18S-5.8S-26S (35S) ribosomal DNA *loci*. The data have been obtained from >600 publications on plant molecular cytogenetics, mostly based on fluorescent *in situ* hybridization (FISH). This edition of the database contains information on 1609 species derived from 2839 records, which means an expansion of 55.76 and 94.45%, respectively. It holds the data for angiosperms, gymnosperms, bryophytes and pteridophytes available as of June 2013. Information from publications reporting data for a single rDNA (either 5S or 35S alone) and annotation regarding transcriptional activity of 35S *loci* now appears in the database. Preliminary analyses suggest greater variability in the number of rDNA *loci* in gymnosperms than in angiosperms. New applications provide ideograms of the species showing the positions of rDNA *loci* as well as a visual representation of their genome sizes. We have also introduced other features to boost the usability of the Web interface, such as an application for convenient data export and a new section with rDNA–FISH-related information (mostly detailing protocols and reagents). In addition, we upgraded and/or proofread tabs and links and modified the website for a more dynamic appearance. This manuscript provides a synopsis of these changes and developments.

**Database URL:** http://www.plantrdnadatabase.com

## Introduction

The Plant rDNA database (www.plantrdnadatabase.com) is an online and open access resource providing information about the numbers, positions and organization of ribosomal DNA *loci* in plant species based on data reported in published papers on plant molecular cytogenetics. It now includes information from papers already published or in press up to June 2013 on angiosperms, gymnosperms, bryophytes and, for the first time, on pteridophytes.

Ribosomal RNAs are fundamental components (both structurally and functionally) of virtually all cell types. In most plants, 5S and 18S-5.8S-26S rDNA (35S) rDNAs are present in high copy numbers to meet the high cellular requirement for ribosomes. They are repetitive, tandemly arranged and clustered at different chromosomal *loci,* i.e. there is typically a spatial separation between the 5S and 18S-5.8S-26S clusters on chromosomes (1). However, recent evidence shows that the four genes can also be linked in a single unit (18S-5.8S-26S-5S) in plant groups as diverse as the genus *Artemisia* and some closely related Asteraceae (2–4) in angiosperms, in gymnosperms such as *Ginkgo* or *Podocarpus* (5, 6) and even in some bryophytes (7–9). Owing to the development of *in situ* hybridization techniques with rDNA probes, visualization of rDNA *loci* directly on the chromosomes of thousands of plant species and determination of their number, placement and organization has become possible. Although the earliest *in situ* experiments used radioactive labelling of probes (10, 11), the vast majority of papers interested in these characteristics are based on fluorescent *in situ* hybridization (FISH) with rDNA probes.

Sequences of ribosomal RNA genes and their spacers have been used profusely in an array of molecular phylogenetic (12, 13) and other approaches to plant evolution (14–16). Number, position and rDNA organization can also be used for, or at least complement comparative, evolutionary and systematic approaches (17–19), assuming that the karyotypes of closely related species are likely to be more similar than those of distant species (20, 21). The typical application scenario would be that of scientists interested in the molecular cytogenetics of a particular group of plants. First, they would need to know if there are any published results on their candidates at any taxonomic level. If this was so, a query to the database would provide information on the rDNA cytogenetic pattern, which could serve as a guide for future *in situ* experiments or could provide a background in which to analyse or discuss their data. These applications may include studies in crop science, plant breeding, evolutionary biology or systematics (22, 23). In the era of molecular phylogenetic and next-generation sequencing methods, cytogenetic data are useful because unique information on genome organization at the chromosome level can be obtained, from basic chromosome identification to the spatial location of specific, single or low-copy sequences, information otherwise difficult to obtain.

With this premise, we enabled the systematic arrangement of this information by constructing the Plant rDNA database (release 1.0). In addition to the raw data on the numbers, positions and linked/unlinked arrangement of rRNA genes, the resource also provides chromosome number, ploidy level, genome size and life cycle (where available), although DNA sequence information is not supplied. A complete description of the contents and operation of the database can be found in (24). At the time of its first release no initiatives known to us offered this kind of information, preceding publication of a paper (25) that statistically analysed 35S rDNA *loci* with respect to their chromosomal position for 846 species. The incessant growth in information on this topic and the positive reception of our Web page triggered the second release only two and a half years after its launch. Owing to its release, the database has received around 4000 visits, with a mean number of 150 visits per month from the beginning of 2014. Of these, ∼65% are from unique visitors. Several recent research works have used and cited the resource (25–30). The aim of this article is to describe the updated data content that may aid in improving our understanding of the evolution of plant ribosomal DNA *loci* numbers and distributions (Garcia *et al.* unpublished results), as well as to explain the new features and improvements made to the database Web environment.

## Database content update: 600 publications milestone passed

Two and a half years after the first release of the database, the overall database content has increased by 94.45% in terms of records (from 1460 to 2839) and by 55.76% in terms of species (from 1033 to 1609). The number of publications recorded has almost doubled from 319 to 610 papers, (a 91.22% increase), not only because the scientific interest in the subject remains strong, but also because of the inclusion of papers reporting information for a single rDNA. The first release compiled papers reporting chromosomal positions of both 35S and 5S genes, whereas studies on solitary genes were neglected. Moreover, some of our users had also suggested the inclusion of (and sometimes even submitted) data on a single rDNA type, as described for the orchid genus *Cymbidium* (31), among others. Considering this, the present release includes data from 33 papers on 5S rDNA only and 129 papers on 35S rDNA only. Many of them have been particularly difficult to obtain because of their age or to publication in journals of restricted/local distribution, adding to the value of the database. Additionally, all publications were reviewed to extract information about the transcriptional activity of the 35S *loci*, noting the presence of secondary constrictions, satellites or nucleolus organizer regions (NORs) whenever the publication stated this, i.e. if silver nitrate staining was performed, or when the satellites or secondary constrictions were evident in the chromosome spreads. The methodology followed for the exhaustive paper search is the same as described in (24).

**Figure 1.**
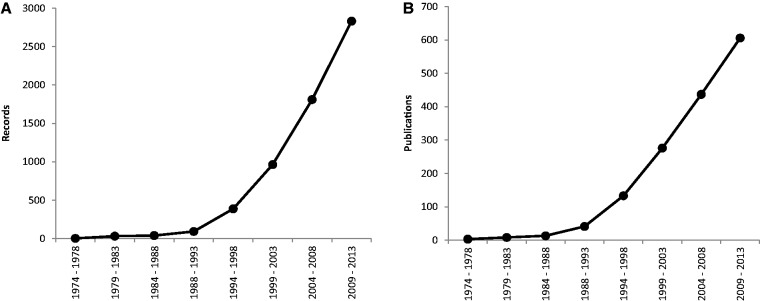
Mean number of records (**A**) and publications (**B**) reported per year over eight successive 5-year periods, between 1974 and 2013. Data taken from the Plant rDNA database (Release 2.0, July 2013).

Since 1974, the rate of production of new data has steadily increased; its expansion was most pronounced in the period 1994–1998 (Figure 1). Furthermore, in past 5 years we have witnessed a steady increase of new publications and records on plant cytogenetics based on rDNA FISH (169 new publications containing 1021 new records), which necessitated the first update after the relatively recent release of the database in 2011. The majority of estimates are for angiosperms (2713 records from 562 source references), with the others comprising 115 for gymnosperms (corresponding to 42 references), six for pteridophytes (from three references) and five for bryophytes (also from three references).

**Figure 2.**
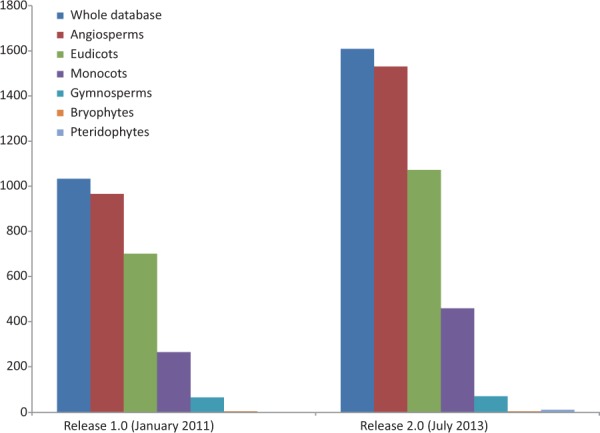
Growth of the database in terms of the number of species represented: the whole database expanded from 1033 to 1609 (55.76%), angiosperms from 966 to 1531 (58.49%) of which eudicots increased from 701 to 1072 (52.92%), monocots from 265 to 459 (73.20%) and gymnosperms from 64 to 69 (7.81%). Bryophyte species recorded did not increase and pteridophytes are represented for the first time in the database, with data for six species. Data taken from the Plant rDNA database (Release 2.0, July 2013).

Represented genera have grown in number from 245 to 351 (43.27%) and families from 60 to 84 (40%). We provide evidence for a new plant division, Pteridophyta, and for the large plant groups species have expanded our records by 58.49% for angiosperms (52.92% for eudicots and 73.21% for monocots) and 7.81% for gymnosperms (Figure 2). Some well-known angiosperm families for which there was no prior record (Betulaceae, Oleaceae, Theaceae, Vitaceae and others) are now included, and records for the huge family Orchidaceae have grown significantly (from 6 to 59). With respect to the previous release, the five most widely assessed families are still the same, although data for Poaceae have been particularly enriched, and it is now the best represented family (Table 1). However, many well-known families such as Lauraceae or Ericaceae still lack data or are poorly represented in the database.

**Table 1.** A comparison of the number of records for the most popular and widely represented families in the Plant rDNA database.

Family	Release 1.0	Release 2.0	Increase (%)
Poaceae	204 (2)	551 (1)	170
Asteraceae	157 (3)	356 (2)	127
Fabaceae	207 (1)	331 (3)	60
Brassicaceae	135 (4)	161 (4)	19
Solanaceae	86 (5)	138 (5)	60

The ranking of the best represented plant families is in brackets.

## rDNA loci number diversity

**Figure 3.**
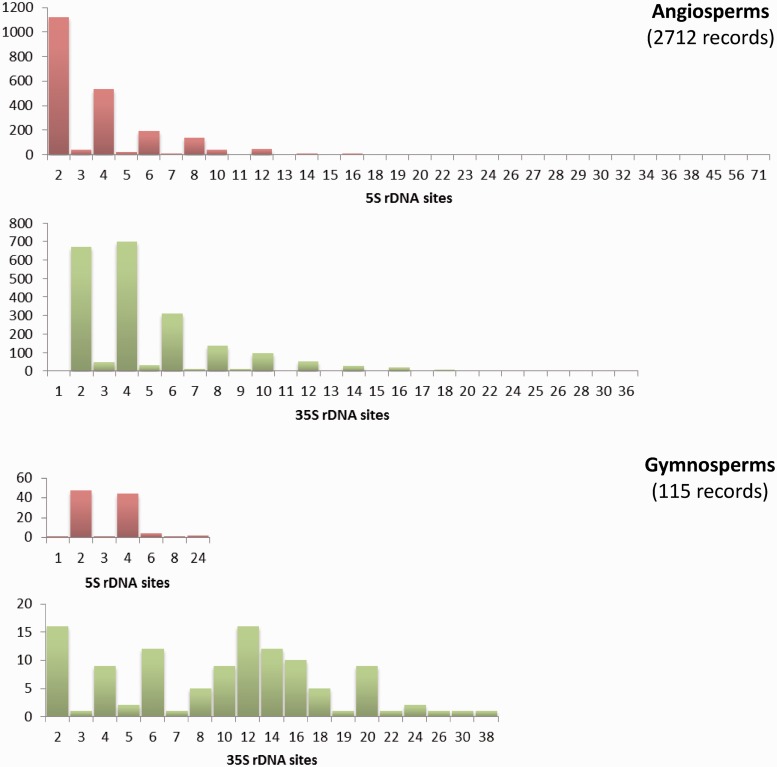
Histograms showing the distributions of the number of 5S and 35S rDNA *loci* in angiosperms and gymnosperms. Graphs are not at the same scale, and all numbers in the X axis with no or small bars represent values with little presence, as the scale of the graphs does not allow their depiction. Numbers of chromosomes represent counts at metaphase. Data taken from the Plant rDNA database (Release 2.0, July 2013).

The Plant rDNA database can provide an overview of the distribution of 5S and 35S rDNA *loci* numbers across angiosperms and gymnosperms (data available in the remaining groups remain too limited for this purpose). There are few cases of exceptionally high numbers of sites in both groups; however, low numbers of 5S and 35S sites are the rule (Figure 3). The exception is the number of 35S *loci* in gymnosperms whose distribution is particularly heterogeneous. Such differing profiles are probably an expression of the contrasting genome dynamics operating between the two plant lineages. This supports the need to continue compiling and analysing numbers and distributions of rDNA *loci* across the plant tree of life to obtain a global view of the evolution of these repetitive DNA sequences in plants.

## Web interface improvements

**Figure 4.**
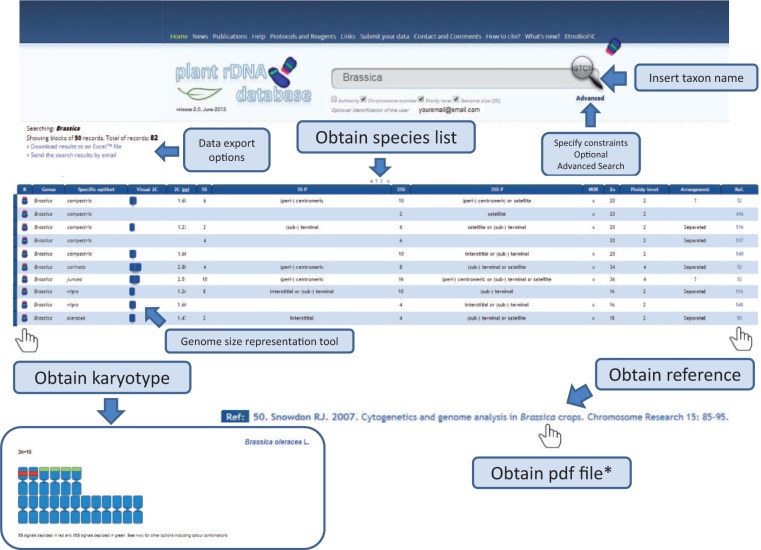
Flowchart showing the possible database outputs and options for a given search. The karyotype appears as a pop-up window, in which the 5S rDNAs are depicted in red and the 35S in green.

Release 2.0 of the Plant rDNA database includes several advances that may enhance the user’s experience. We introduced karyotype and genome size representation tools that allow visualization of 5S and 35S rDNA *loci* in a standardized ideogram, and a bar representing species genome size. In these graphs, chromosomes are all depicted as being metacentric with red and green bands (corresponding to the number of 35S and 5S signals, respectively) located interstitially, (peri-) centromerically or (sub-) telomerically. Exceptions such as linked 5S and 35S rRNA genes in the same unit, among others, are also considered and differentially marked, which is explained in a short tutorial. The ideogram may be accompanied by a bar representing genome size with an indication of the mean genome size of the genus (or higher taxonomic group if required). This allows users to quickly identify genome size differences within a given search output. An example on how these tools work is shown in Figure 4.

Another novel feature of Release 2.0 is that all data are available for bulk download. A small application in the search page allows immediate data export as Excel™ or CSV files (comma-separated values, which store tabular data in plain-text form), as well as direct email of the results of a search.

## New and upgraded page tabs

Two new page tabs have been created for the present site release. The first, ‘Protocols and Reagents’, contains our own protocols, reagents and checklists for performing FISH of rRNA genes in plants, including the preparation of chromosome spreads and probe labelling. There are also other useful links to companies providing the required products as well as directions to the FISH protocols, tips and/or troubleshooting guides of others. Citations of the most relevant books on *in situ* hybridization and information on where to purchase them are listed at the bottom of the page. With this, we aim to promote and ease the performance of the rDNA FISH method among researchers interested in plant cytogenetics. The second new page tab, entitled ‘What’s new?’, will describe and explain new developments and updates in future releases.

Concerning the upgraded page tabs, ‘Home’ has been renewed, with updated and more accessible text that briefly states the intended purpose of the database. A graphic depiction of the database resources for different plant groups is also included, along with graphics showing the increase in data from the first to the second release. Additionally, the ‘Learn more’ section has been enhanced to include more complete, visual information about rRNA genes and FISH. To illustrate to the user the range of information available in the database, we have composed a continuous series of plant pictures (of commonly identifiable plants) with data on their rDNA *loci* and chromosome number.

Additionally, ‘Links’ has been expanded to include other interesting sites, as well as having been revised for broken hyperlinks and removed content. Likewise, in ‘Publications’ the list has been expanded to include the 291 new references, each with their own direct hyperlink to the ‘pdf’ file (accessible if the user/user’s institution has permission—otherwise only the abstract will be shown) in the majority of cases; however, access to some publications is still restricted to the hard copy format. References previously listed in this page tab have also been reviewed, updated or corrected when necessary.

Both ‘Contact & Comments’ and ‘Submit your data’ have been improved to allow better feedback and data submission, including an updated submission form for the latter. Researchers are encouraged to send in their data even if it is unpublished as these will not be shown in the database, although a ‘publication pending’ message will appear when entering the species name until the final release of their publication. Authors are asked to accompany their data submission with the corresponding publication or at least with high quality pictures attesting the information. Any query from the users will be answered within a short period, normally one to two working days, and comments, corrections and suggestions are particularly welcome.

The tab ‘Help’ comprises a short explanation of the karyotype and the genome size representation tools as well as some new notes regarding interaction with the database. Finally, in ‘How to cite’ new instructions are given for citing each release, and the database release article (24) is included and available for direct download (provided that the user has access, as stated previously).

## Other modifications

Additional minor changes that may increase the quality of a visit to our database are an improved, enhanced and debugged browsing environment, a change of font and a slight change of the design of the Web page. Within a given search output, the user can now directly download the source publication (or access its abstract) by clicking on the reference number, without needing to consult the ‘Publications’ tab. The advanced search has been simplified so that it is easier to use. To better inform us of the profiles and preferences of our users for the planning of future improvements, we have included optional user identification, as well as the automatic logging of each search.

## Conclusions and Future Developments

The second release of the Plant rDNA database represents a considerable effort in compilation, as the number of records and source publications consulted has in a short period almost doubled with respect to the first one. At present, the database is the most comprehensive resource available for information on 5S and 35S rDNA *loci* numbers, positions and structures in plants (with no equivalent in animals). The numerous improvements and extensions made may boost the Web interface functionality, but in this release we were particularly interested in emphasizing the expansion in technical information as well as being more user-friendly. The huge increase in available data observed in only 2 years foments our determination to perform regular (and more frequent) content updates so records of the global knowledge of plant rDNA *loci* distribution stay up-to-date. In the coming years, we also intend to include data on chromosome morphology and a number of active NORs, whenever the source publication provides this information. With the whole genome sequencing of more and more plant species, it may also be possible to indicate which of those listed in the database are being or have been fully sequenced.
